# Supplementary material for: Early life factors documented in electronic health records predict recurrent acute otitis media
Source: PLoS One. 2026 Sep 18;21(9):e0345434. doi: 10.1371/journal.pone.0345434 (PMC13588358; doi:10.1371/journal.pone.0345434)
Supplement: Supplementary file 1 [file pone.0345434.s001.docx]

| **S1 Table.** Computable phenotypes for AOM and relevant maternal and infant comorbidities | | |
| --- | --- | --- |
| **Condition** | **ICD-9 codes** | **ICD-10 codes** |
| AOM episode | 328* | H65.0-H65.199, H66.00-H66.019, H66.4*, H66.9* |
| Cleft lip and/or palate | 749*,744* | Q37*, Q16*, Q17* |
| Trisomy 21 | 758* | Q90* |
| Immune deficiency | 279* | D80*, D81*, D82*, D83*, D84* |
| Maternal Group B Strep colonization | V02.51 | O99.820, Z22.330 |
| Maternal fever during delivery | 659.20, 659.21, 659.23 | O75.2, O75.3 |
| Chorioamnionitis | 658.4* | O41.12* |
| Gestational diabetes | 648.8 | O24.0* – O24.9* |
| Maternal tobacco use during pregnancy | 649.0* | O99.33* |
| Atopic disease | 372.05, 372.14, 477*, 691*, 995.6*, 995.7, V15.0* | H10.1*, H10.45, J30*, L20*, T78*, Z91.0* |
