# Supplementary material for: Early life factors documented in electronic health records predict recurrent acute otitis media
Source: PLoS One. 2026 Sep 18;21(9):e0345434. doi: 10.1371/journal.pone.0345434 (PMC13588358; doi:10.1371/journal.pone.0345434)
Supplement: Supplementary file 2 [file pone.0345434.s002.docx]

| **S2 Table.** Time between AOM episodes in sporadic and recurrent AOM | | | | | | |
| --- | --- | --- | --- | --- | --- | --- |
|  | **Episode 1 to 2** | | **Episode 2 to 3** | | **Episode 3 to 4** | |
|  | **Sporadic AOM** | **Recurrent AOM** | **Sporadic AOM** | **Recurrent AOM** | **Sporadic AOM** | **Recurrent AOM** |
| Patients | N = 2788 | N = 1373 | N = 1284 | N = 1373 | N = 510 | N = 454 |
| Mean (sd) | 300.9 (294.8) | 97.0 (112.9) | 327.9 (267) | 88.5 (105.5) | 313.1 (248.2) | 81.5 (83.1) |
| Median (IQR) | 197.5 (83.8, 414.2) | 62 (31, 110) | 257 (130.5, 440) | 57 (31, 105) | 240.5 (133, 420.2) | 54 (29, 101.8) |
